# A System of Human Anatomy General and Special

**Published:** 1847-05

**Authors:** 


ART. VII.—*A System of Human Anatomy, General and Special.* By ERASMUS WILSON, M.D., Lecturer on Anatomy in London. Third American from the third London edition. Edited by PAUL B. GODDARD, A.M , M.D., Professor of Anatomy in the Franklin Medical College of Philadelphia. With 233 Illustrations by Gilbert. 8vo. pp. 610. Philadelphia: Lea and Blanchard: 1847.

This beautiful work, it will be seen from the title-page, has already reached the third edition in this country, although it is but a little while since the first was issued. It needs no commendation from the press; its character is established.

